# Signaling Pathways Involved in Renal Oxidative Injury: Role of the Vasoactive Peptides and the Renal Dopaminergic System

**DOI:** 10.1155/2014/731350

**Published:** 2014-11-11

**Authors:** N. L. Rukavina Mikusic, M. C. Kravetz, N. M. Kouyoumdzian, S. L. Della Penna, M. I. Rosón, B. E. Fernández, M. R. Choi

**Affiliations:** Department of Pathophysiology, Faculty of Pharmacy and Biochemistry, University of Buenos Aires, CONICET, INFIBIOC, 1113 Buenos Aires, Argentina

## Abstract

The physiological hydroelectrolytic balance and the redox steady state in the kidney are accomplished by an intricate interaction between signals from extrarenal and intrarenal sources and between antinatriuretic and natriuretic factors. Angiotensin II, atrial natriuretic peptide and intrarenal dopamine play a pivotal role in this interactive network. The balance between endogenous antioxidant agents like the renal dopaminergic system and atrial natriuretic peptide, by one side, and the prooxidant effect of the renin angiotensin system, by the other side, contributes to ensuring the normal function of the kidney. Different pathological scenarios, as nephrotic syndrome and hypertension, where renal sodium excretion is altered, are associated with an impaired interaction between two natriuretic systems as the renal dopaminergic system and atrial natriuretic peptide that may be involved in the pathogenesis of renal diseases. The aim of this review is to update and comment the most recent evidences about the intracellular pathways involved in the relationship between endogenous antioxidant agents like the renal dopaminergic system and atrial natriuretic peptide and the prooxidant effect of the renin angiotensin system in the pathogenesis of renal inflammation.

## 1. Introduction

A normal redox state of cells depends on a delicate balance between oxidative species and antioxidant mechanisms. Acting as cellular messengers, reactive oxygen species (ROS) are involved in the destruction of invading pathogens [1]. Chronic inflammatory conditions such as atherosclerosis or hypertension can alter the normal redox state of the cells through an overproduction of free radicals that leads to an increase in oxidative stress with disruption of the normal cellular signaling mechanisms [2–5]. In the kidney, oxidative stress and infiltration of inflammatory cells represent key factors for the development of renal injury and hypertension [6]. Angiotensin II (Ang II) that displays hypertensive and prooxidant properties, by one side, and the atrial natriuretic peptide (ANP) and renal dopamine, by the other side, both with hypotensive and antioxidant properties, are local factors closely related to the development and progression of glomerular and tubular injury [7, 8].

## 2. Renin-Angiotensin System and Renal Oxidative Stress

**Figure 1:**
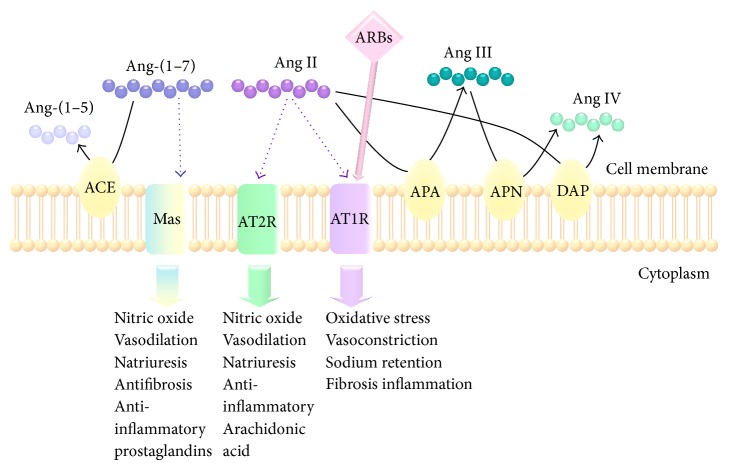
Metabolism and major functions of angiotensin peptides. Angiotensin-(1–7) is metabolized by angiotensin-converting enzyme (ACE) to form Ang-(1–5). Angiotensin II (Ang II) is catabolized by aminopeptidase A (APA) to form angiotensin III, which is further hydrolyzed by aminopeptidase N (APN) to form angiotensin IV. Also, Ang II can be directly cleaved by dipeptidyl aminopeptidase IV (DAP) to produce Ang IV. Ang-(1–7) binds to Mas receptor to exert anti-inflammatory effects. With author's permission (S. L. Della Penna).

Ang II mediates most of the renin angiotensin system (RAS) effects through activation of two types of receptors: Ang II type 1 (AT1R) and Ang II type 2 (AT2R). In the last decades, novel components of the RAS have been recognized, including the (pro) renin receptor, the angiotensin-converting enzyme type 2 (ACE-2)/Ang (1–7)/Mas axis, and other Ang peptides (Ang III, IV, 1–5) (Figure 1) [9]. The observation that angiotensinogen, renin, ACE, and AT1R are expressed in multiple tissues suggests the existence of multiple local RAS, acting as independent entities from the systemic RAS [10]. In particular, there is a local renal RAS that synthesizes and secretes Ang II, reaching a concentration 100-fold higher in the lumen than in the plasma [11–13].

The finding that intrarenal Ang II content is elevated in many forms of hypertension supports the idea that the intrarenal RAS plays a crucial role in the development of hypertension and the RAS-associated injury [14]. Furthermore, renal RAS overactivity is associated with the development of diverse pathological processes in the kidney, including glomerular sclerosis, diabetic nephropathy, and renal artery stenosis [15, 16]. Indeed, experiments in gene-targeted mice demonstrated that the elevation of the locally produced renal Ang II induced the elevation of blood pressure, together with the development of renal inflammation and fibrosis [11, 17]. In this way, the use of ACE inhibitors and AT1R blockers is recommended as first-line therapy in hypertensive patients with renal disease (Figure 1) [10]. These pharmacological agents attenuate renal disease in both preclinical and clinical studies and are effective and well tolerated, and in addition they improve morbidity and mortality associated with cardiovascular events [18, 19]. Therefore, in pathological conditions, Ang II may contribute to impairing renal function by inducing oxidative stress, inflammation, and fibrosis and downregulation of water channels aquaporins [12, 20–26]. It must be pointed out that these processes could be detected before the elevation of blood pressure and even when the systemic RAS was inhibited [11, 26].

Besides the systemic and the local tissue RAS, the presence of an intracrine or intracellular RAS has been recently described [27]. Synthesized Ang II within the cell binds to intracellular and/or nuclear AT1R, AT2R, or Mas receptors and activates downstream signalling pathways. By this way, intracellular Ang II can induce cellular or nuclear responses independently of its cell surface receptors [27–30]. These and other findings provided new knowledge about novel roles of the RAS in many fields, beyond blood pressure control.

In the kidney, AT1R mediates vasoconstriction of glomerular microvasculature, modifying the glomerular filtration rate, the tubuloglomerular feedback, and cell growth [10]. Moreover, Ang II modifies, through this receptor, the activity of different transporters in the kidney such as Na^+^/H^+^ exchanger (NHE), epithelial sodium channels (ENaCs), Na-K-2Cl cotransporter (NKCC2), and Na-Cl cotransporter (NCC) [10, 31]. Overall, these actions contribute to an increased capacity of the kidney to preserve sodium and maintain blood pressure within normal values. The AT2R is localized in glomerular epithelial cells, proximal tubules, collecting ducts, and renal vasculature. It is considered as a functional antagonist of AT1R and is associated with vasodilation, apoptosis, antiproliferation, and increase of natriuresis, by stimulation of nitric oxide/cGMP/protein-kinase G (PKG) pathway [32]. Activation of AT2R induces stimulation of endothelial nitric oxide synthase (eNOS) to produce nitric oxide (NO) that is a potent vasodilator molecule and essential for the maintenance of cardiovascular health [33].

The AT1R is a member of the G protein-coupled receptor family associated with Gq/11, Gi, G_12_, and/or G_13_ [34]. Ang II binding to AT1R leads to activation of phospholipase C (PLC), A2 (PLA_2_), or D (PLD), which ultimately raise intracellular second messengers inositol-1,4,5-triphosphate (IP_3_), diacylglycerol, arachidonic acid, Ca^2+^, and ROS concentrations [35]. These signaling factors subsequently activate Rho kinase and mitogen-activated protein kinases (MAPK) including p44/42 MAPK, p38 MAPK, and c-jun N-terminal kinase [36, 37]. However, G protein independent pathways can also play a crucial role on the development of pathophysiological processes. In this way, it has been recently demonstrated that Janus kinase (JAK) is elicited by Ang II as a key target in the development of RAS-associated diseases like hypertension [38]. On the other hand, the RAS and inflammatory cytokines can synergize their effects to elevate blood pressure [13]. Circulating Ang II, as well as locally produced Ang II, can increase cytokine levels, including interleukin 6 (IL-6) and interferon *γ* (IFN-*γ*), which in turn synergize the activation of Janus kinase/signal transducers and activators of transcription (JAK/STAT) pathway. This cascade of events is finally a potent stimulus for angiotensinogen expression and thereby contributes to increase the local synthesis of Ang II and the progression of hypertension and the establishment of renal injury.

**Figure 2:**
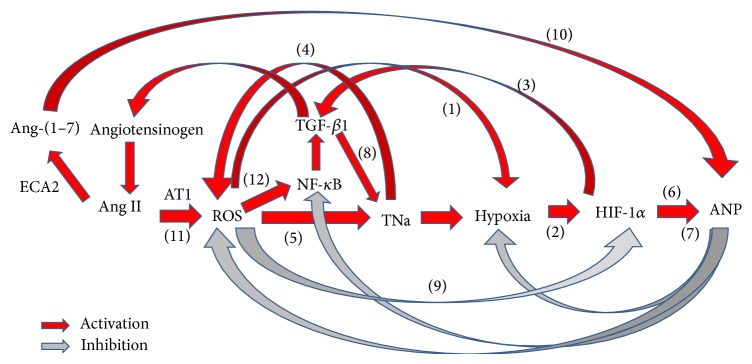
Interaction between renin angiotensin system and oxidative stress. (1) ROS can activate the mitochondrial uncoupling protein 2 (UCP-2), leading to inefficient renal O_2_ usage and contributing to renal hypoxia. (2) Hypoxia induces the transcription factor hypoxia-inducible factor-1*α* (HIF-1*α*) which coordinates the expression of diverse adaptive genes against the hypoxic injury. (3) HIF-1*α* transcriptionally upregulates the expression of soluble growth factors (TGF-*β* and VEGF). (4) Increased sodium tubular transport, luminal flow, or cytokines release upregulates NADPH oxidase, which produces ROS. (5) Superoxide anion increases Na-K-2Cl cotransport activity in the thick ascending limb, enhancing further oxidative stress. (6) Hypoxia regulates ANP. (7) ANP in addition to its natriuretic actions exerts protective effects on several cell types in response to the oxidative stress and fibrosis and on the adaptation to hypoxia. (8) TGF-*β*1 upregulates the transcription of serum and glucocorticoid-dependent kinase hSGK1, involved in the regulation of two important factors for cell volume regulation, as the renal epithelial Na^+^ channel ENaC and the thick ascending limb Na^+^, K^+^, 2Cl^−^ cotransporter NKCC. (9) ROS, especially superoxide, degrade HIF-1*α* by activating ubiquitin-proteasome and thereby decrease the activation of many oxygen-sensitive genes. (10) Angiotensin-converting enzyme 2 regulates renal ANP through angiotensin-(1–7). (11) The signal transduction of Ang II through AT1R enhances ROS production. (12) NADPH oxidase contributes to renal damage through NF-*κ*B.

ROS play an important pathophysiological role in the kidney [33, 39–41]. Nicotinamide adenine dinucleotide phosphate reduced form (NADPH) oxidase is a predominant enzyme involved in the development of oxidative stress; it is upregulated by the increase of sodium tubular transport, or luminal flow, or by cytokines release [42, 43]. ROS production activates several intracellular signalling mechanisms, such as AP-1 (activator protein-1) and NF-*κ*B (nuclear factor-*κ*B) transcription factors [44, 45]. When Ang II binds to AT1R, it stimulates NADPH oxidase, magnifying the production of ROS, such as superoxide anion (O_2_^−^). In turn, the superoxide anion increases tubular NaCl transport, enhancing further the oxidative stress. ROS can also activate the mitochondrial uncoupling protein 2 (UCP-2), leading to an inefficient use of renal O_2_ and contributing to renal hypoxia [46]. Changes in cellular oxygen concentrations induce the response of tightly regulated pathways that attempt to restore oxygen supply to cells and modulate the cell function under hypoxic conditions. Most of these responses are mediated by the induction of the transcription factor hypoxia-inducible factor-1*α* (HIF-1*α*) which coordinates the expression of diverse adaptive genes against the hypoxic injury [47, 48]. HIF-1*α* is stabilized by the sum of opposed effects: by one hand, the increase of sodium transport by the tubules generates relative hypoxia into the cells, thereby increasing HIF-1*α*; but on the other hand, HIF-1*α* can be diminished by the oxidative stress, induced by the increase in sodium transport. Even though the oxidative stress diminishes the efficiency of the use of oxygen, thereby increasing hypoxia, it also increases the proteasomal degradation of HIF-1*α*, contributing to its catabolism and diminishing its concentration. HIF-1*α* upregulates the transcription of metabolic proteins (GLUT-1), adhesion proteins (integrins), soluble growth factors (TGF-*β* and VEGF), and extracellular matrix components (as type I collagen and fibronectin), which enhance the repair process. For these reasons, HIF-1*α* is considered as a positive regulator of organ repair and tissue fibrosis [49, 50]. It is known that transforming growth factor-beta1 (TGF-*β*1) upregulates the transcription of the serum and glucocorticoid-dependent kinase hSGK1, involved in the regulation of two important factors for cell volume regulation, as the renal epithelial Na^+^ channel ENaC and the thick ascending limb Na^+^, K^+^, 2Cl^−^ cotransporter NKCC [51]. The increase of cell volume stimulates protein synthesis and inhibits protein degradation, contributing to enhancing the net formation and deposition of matrix proteins. In addition, TGF-*β*1 transduces intracellular signals through type 1 (TGF-*β*R1) and type 2 (TGF-*β*R2) receptors, via the nuclear translocation of Smad3 proteins, thus contributing to a fibrotic response [52]. Recent studies show that TGF-*β*1 stimulates angiotensinogen gene expression and its action through ROS generation, p38 MAPK activation, and p53 expression [53]. Ang II directly stimulates nuclear AT(1a) receptors and markedly increases* in vitro* transcription of mRNAs for TGF-*β*1 [54]. These mechanisms suggest that Ang II and TGF-*β*1 may form a positive feedback loop to enhance their respective gene expression leading to renal injury. Ang II also induces NF-*κ*B subunit p65 phosphorylation and its nuclear translocation [55]. NF-*κ*B activates genes involved in the inflammatory and fibrotic responses, which are characterized by infiltration of monocytes/macrophages and lymphocytes and upregulation of Ang II and other proinflammatory factors such as adhesion molecules (V-CAM 1 and I-CAM 1), chemokines (MCP-1 and RANTES), and cytokines (TGF-*β*1) [9, 56–58]. Furthermore, gene silencing of ILK (integrin-linked kinase) prevented the upregulation of NF-*κ*B-related proinflammatory gene, demonstrating that ILK plays a pivotal role in the modulation of inflammation by regulating the NF-*κ*B pathway and suggests a potential therapeutic target to be used in the inflammatory renal disease produced by Ang II. In addition, intrarenal Ang II production is highly stimulated by the excess of salt in the diet [10, 21, 59]. A renal proinflammatory response, secondary to the excess of sodium, favours further sodium retention and thus the development of arterial hypertension [21, 60, 61]. The molecular mechanisms of the inflammatory response to salt-sensitive hypertension remain to be completely characterized. The increase in sodium reabsorption in the renal tubules leads to elevated blood flow and thus to glomerular hyperfiltration. This process augments the metabolic demand of oxygen, which results in a decrease in tissue oxygen tension [62]. The rise in oxygen consumption leads to relative hypoxia that triggers a cascade of events, magnifying ROS production and stimulating the expression of local Ang II and proinflammatory genes (Figure 2).

A growing number of mammalian genes, including ANP, have been identified to play a key role in the cellular adaptive response to counterregulate renal hypoxia and development of fibrosis. In this way, ANP exerts protective effects in several types of cells in response to oxidative stress, fibrosis, and adaptation to hypoxia [63–65].

## 3. Oxidative Stress and Nephroprotective Actions of Atrial Natriuretic Peptide

ANP is a member of the natriuretic peptide family and is synthesized and stored in the atrial myocytes and released in response to the cardiac wall stretching or after endothelin, cytokine, or *α*-adrenergic stimulation [66–68]. ANP can regulate blood pressure and volume homeostasis by its natriuretic, diuretic, vasodilator, and renin-angiotensin-aldosterone suppressor activities [69]. It has been demonstrated that ANP can also act as an autocrine/paracrine factor in several organs, such as kidney, lung, thymus, and liver [70–73]. In fact, many studies have proposed the kidney as a source of ANP [74, 75]. All these findings together suggest new biological actions of ANP besides its classical natriuretic, diuretic, and vasodilator effects. ANP exerts these actions by its binding to specific receptors. There are three classes of natriuretic peptide receptors identified (NPRA, NPRB, and NPRC) usually located on the plasma membrane of target cells [76, 77]. In the kidney, mRNA and protein expression of both NPRA and NPRC receptors have been demonstrated [78–80]. ANP exerts its main biological actions by binding to NPRA, which has intrinsic guanylate cyclase (GC) activity. Besides the GC domain, it also contains an extracellular binding domain (ECD) and a preserved intracellular kinase homology domain (KHD). Activation of the intrinsic GC domain by ANP binding to the ECD increases intracellular guanosine 3,5-cyclic monophosphate (cGMP) levels, which has three known cGMP binding proteins: cGMP-dependent protein kinases (PKG), cGMP-binding phosphodiesterases (PDEs), and cyclic nucleotide-gated (CNG) ion channels [81]. The activation of PKG triggers different events related to cell growth, apoptosis, proliferation, and inflammation [82]. PDEs degrade cyclic nucleotides into inactive 5′-nucleotide monophosphates, being crucial regulators of cyclic nucleotide signalling [81]. NPRC exists as a homodimer linked by disulfide bonds and has a single transmembrane domain, an ECD homologous to NPRA, and a short cytoplasmic domain [83]. NPRC was first considered as a clearance receptor without any physiological actions, playing its role during internalization and removal of the hormone from the circulation [84]. However, it has been proved that NPRC can elicit physiological functions by its coupling to adenylyl cyclase (AC) inhibition through an inhibitory guanine nucleotide-regulatory protein (Gi) and/or by activation of PLC [85]. The model of NPRC gene (Npr3) knockout mice showed that both homozygotes and heterozygotes mice have altered cardiovascular and renal functions, with reduced ability to concentrate urine, mild diuresis, blood volume depleted, and blood pressure values below normal levels. These changes suggest that NPRC would modulate the local availability of natriuretic peptides, allowing them to response to specific local needs. Moreover, homozygotes had skeletal deformities, pointing out the importance of these peptides in the process of bone formation [86].

Besides its role in the regulation of blood pressure and volume homeostasis, it has been reported that ANP can exert protective effects in response to oxidative stress, fibrosis, and the adaptation to hypoxia [63–65]. However, little is known about the role exerted by ANP in the kidney in such states. In this sense, Koga et al. showed that ANP has antioxidant effects in rats, as demonstrated by its ability to attenuate ROS levels in a renal ischemia-reperfusion injury model [87]. In another study, Finch et al. showed that, in uremic rats, the progression of renal worsening and oxidative stress was accompanied by the increase in 133-fold of ANP mRNA in tandem together with an increase in blood pressure, suggesting that ANP mRNA increase would act as a protective mechanism [88]. Several experimental data show that hypoxia is an independent factor that regulates the synthesis and release of natriuretic peptides. It has been reported by Chun et al. that, in cultured atrial myocytes without any stretch changes, hypoxia stimulated the ANP gene expression, enabling the localization and characterization of a 120 bp region of hypoxia-response elements within the ANP gene promoter [89]. In this sense, Della Penna et al. demonstrated a significant increase in HIF-1*α* expression associated with augmenting intrarenal ANP levels, suggesting a potential adaptive mechanism by which intrarenal ANP levels would rise in response to the oxidative stress produced in the kidney of rats chronically fed with a high sodium diet [90].

**Figure 3:**
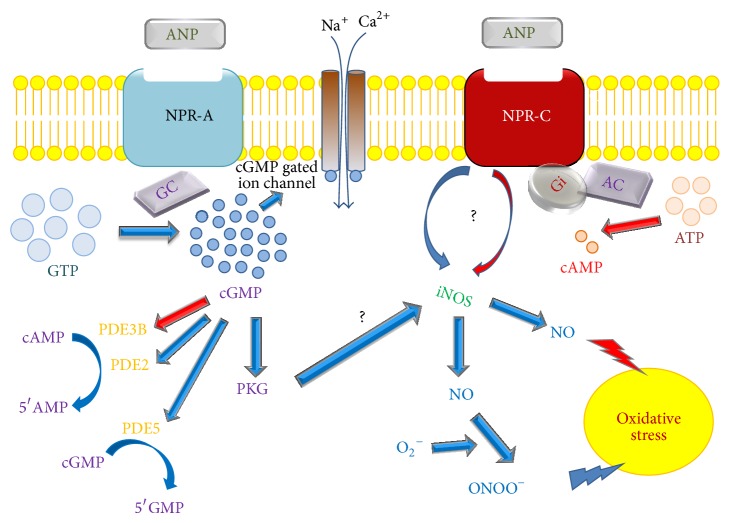
Effects of ANP on NOS activity in the kidney. NO synthesis can be stimulated by ANP via NPRA receptors. It has also been reported that NPRC natriuretic receptor mediates the activation of NOS by ANP. This evidence is in accordance with studies supporting a protective role of ANP against oxidative stress. In contrast, there is evidence indicating that ANP inhibits the inducible nitric oxide synthase (iNOS) activity via NPRC, leading to the increase of NO levels. It has been shown that an increment in renal NOS activity together with ROS production (represented as superoxide anion O_2_^−^) is able to increase cytotoxic peroxynitrites (ONOO^−^) which in turn reduce NO bioavailability, leading to oxidative stress. The mechanisms by which ANP regulates NOS activity in the kidney are not completely elucidated (blue arrow: stimulatory effect, red arrow: inhibitory effect, question mark: contradictory evidence supporting both stimulatory and inhibitory effects of ANP on NOS activity, and PDE: phosphodiesterase).

On the other hand, it has been shown that an increment in renal NOS activity together with ROS production is able to increase cytotoxic peroxynitrites which in turn reduce NO bioavailability, leading to oxidative stress [91]. There are contradictory evidences regarding the effects of ANP on the activity of the different renal isoforms of NOS (Figure 3). Supporting an antioxidant effect of ANP, McLay et al. have shown that, in cultures of proximal tubular cells, ANP inhibits the inducible nitric oxide synthase (iNOS) activity, via NPRC and cGMP [92]. In addition, Chatterjee et al. showed that, in human proximal tubular cells, ANP inhibited cytokine-induced NO synthesis through a cGMP-independent pathway, involving the NPRC subtype receptor [93]. However, there is also evidence against the described beneficial effects of ANP on oxidative stress. In this way, studies performed in primary cultures of human proximal tubular cells showed that NO synthesis can be stimulated by ANP via NPRA receptors [94]. In addition, it has been reported that the NPRC mediates the activation of NOS by ANP in the kidney (Figure 3) [95, 96]. Regarding this contradictory evidence, de Vito et al. suggested that, in different cell types and systems, ANP could have either antioxidant or prooxidant effects, depending on the experimental conditions and cell context [97]. Like the kidney, the cardiovascular system is another case of conflicting findings. It has been demonstrated in rat aortic smooth muscle cells that physiological concentrations of ANP (10^−10^ to 10^−9^ M) stimulate NPRC and counteract ROS generation by interfering with the lysophosphatidic acid dependent phosphoinositide 3-kinase/ROS/MAPK activation [98]. Moreover, in vascular smooth muscle cells from hypertensive rats, the activation of NPRC by the ring-deleted analog C-ANP4-23 decreased the enhanced expression of Nox4 and p47-phox subunits of NADPH oxidase, removing the enhanced oxidative stress and acting as a potent antioxidant [99]. In contrast, in human umbilical vein endothelial cells, ANP is able to induce mitogen-activated protein kinase phosphatase-1 via the Rac-1 subunit of NADPH oxidase and superoxide generation, suggesting a prooxidant role of ANP [100]. Furthermore, a study carried out in human macrophages showed that ANP was able to induce ROS generation by inhibition of sodium/hydrogen exchanger (NHE) activity and by increasing PLD activity with subsequent activation of NADPH oxidase [101]. Finally, in human endothelial cells, ANP induced the activation of MAPK phosphatase-1 via Rac1 and NADPH oxidase/Nox2 activation [100]. There is no data about the role of ANP on NADPH oxidase activity in the kidney. Therefore, further investigation is needed to complete the understanding of ANP role as nephroprotective agent against oxidative stress.

As a physiological antagonist, ANP may counterregulate RAS activity not only through its natriuretic and vasodilatory properties, but also at different stages of the inflammatory and fibrotic processes [102, 103]. Interestingly, ANP has been shown to regulate eNOS and iNOS activities in blood vessel endothelium, by means of two different pathways: via amelioration of endothelium dependent NO synthesis and also by the concomitant inhibition of cytokine mediated iNOS expression. Moreover, it has been described that ANP inhibits nuclear factor NF-*κ*B activation and inflammatory mediators like iNOS, cyclooxygenase-2, and TNF-*α*, thus decreasing peroxynitrites, cytokines, and chemokines production [73, 104–108]. The increase of mRNA expression of TNF-*α*, IL-1*β*, and IL-6 in the kidney and in the lung was reported in a rat model of renal ischemia-reperfusion injury, with that increment being abolished by pretreatment with ANP [109]. Moreover, Lo et al. demonstrated that endogenous ANP exhibited auto/paracrine effects in renal tubular cells, counteracting the hyperglycemia stimulated by TGF-*β*1 and NF-*κ*B overexpression [110]. ANP also induces the expression of the NF-*κ*B inhibitor I*κ*B, whose transcriptional induction is suggested to be a key step in the inhibitory action of ANP on NF-*κ*B activation [104]. On the other hand, Das et al. demonstrated that renal NF-*κ*B activity increased in the model of NPRA gene knockout mice, stimulating the synthesis of proinflammatory cytokines to produce renal abnormalities. This suggests that NPRA could contribute to counterregulating the effects of NF-*κ*B signaling pathway in the kidney, thus providing renoprotection [111]. All these findings suggest the anti-inflammatory role of ANP, independently of its hemodynamic actions. Likewise, in human proximal tubular cells, ANP inhibits cytokine-induced NO synthesis through a cGMP-independent pathway, involving the NPRC subtype receptor [112]; meanwhile in micro- and macrovascular endothelial cells, ANP inhibits hypoxia-induced inflammatory pathways [73, 112]. Furthermore, it has been demonstrated that the activation of ANP/cGMP/PKG signaling favors the phosphorylation of Smad3 and disrupts TGF-*β*1-induced nuclear translocation of pSmad3 and also, later downstream events, including myofibroblast transformation and the proliferation and expression of extracellular matrix molecules [113]. Rosón et al. [21] have described that a hypertonic sodium overload in normal rats generates a cascade of events in the kidney, including higher sodium tubular reabsorption, which is able to increase oxygen demand, accompanied by an increase in local Ang II, nuclear factor NF-*κ*B activation and TGF-*β*1 expression. In this regard, this early inflammatory response was prevented and reversed by the administration of low and nonhypotensive doses of ANP. This was associated with downregulation of NF-*κ*B and local Ang II, as well as diminished hypoxia evaluated through upregulation of HIF-1*α* expression. Taken together, these results suggest a possible mechanism by which ANP could be acting directly on the inflammatory response inhibiting NF-*κ*B and Ang II and consequently in an indirect way on the inhibition of ROS production, thereby protecting cells from hypoxia and inflammation [21].

## 4. Antioxidant Properties of the Renal Dopaminergic System

The renal dopaminergic system is a local independent natriuretic system necessary to maintain the normal balance of sodium and water, blood pressure levels, and renal redox steady state. Different findings from experimental and clinical studies highlight the participation of renal dopamine in the pathophysiology of renal inflammation, hypertension, diabetic nephropathy, and edema [114–117]. The relationship between dopamine receptors and oxidative stress was not established until the past decade. Although it still remains a subject of intense debate and continuous investigation, different evidences provide a better understanding about the mechanism by which oxidative species and renal dopamine interact at renal tubular level.

Renal locally formed dopamine is a major regulator of proximal tubule salt and water reabsorption and exerts its physiological actions through two different receptors located at the tubular cell surface: D1 receptor subtype (D1R, a member of the D1-like receptor family together with D5R) and D2 receptor subtype (D2R, a member of the D2-like receptor as well as D3R and D4R) [118–121]. By activation of these receptors, dopamine increases renal blood flow and elicits a marked natriuretic response [120–122]. The importance of dopamine as a natriuretic hormone is reflected by its capacity to inhibit sodium transporters almost in the entire nephron [123]. The D1-like receptors, coupled to the stimulatory G proteins G*α*_*s*_ and G_olf_, are characterized by their capacity to activate AC, while D2-like receptors, coupled to the inhibitory G proteins G*α*_*i*_ and G_*o*_, are characterized by their capacity to inhibit AC and modulate ion channels [124, 125]. Although dopamine receptors are abundantly located in the proximal convoluted tubule, dopamine can also induce natriuresis acting on distal nephron segments [126]. The classical signaling pathway for D1-like receptors leads to activation of AC and increases cAMP levels and PKA activation. PKA may either directly phosphorylate a target protein, such as a sodium transporting protein, or initiate a cascade of phosphorylation events by phosphorylation and activation of dopamine and cAMP-regulated phosphoprotein DARPP32 [127]. D1R can also stimulate phospholipase C*β*1 in renal tubules [128]. On the other hand, D2R can suppress Akt (protein kinase B) signaling pathway [129]. Both types of dopamine receptors are also linked to MAPK activation through different pathways and can interact with each other, resulting in new signaling pathways. In renal cortical cells the interaction between D1R and D2R increases PLC stimulation [130].

**Figure 4:**
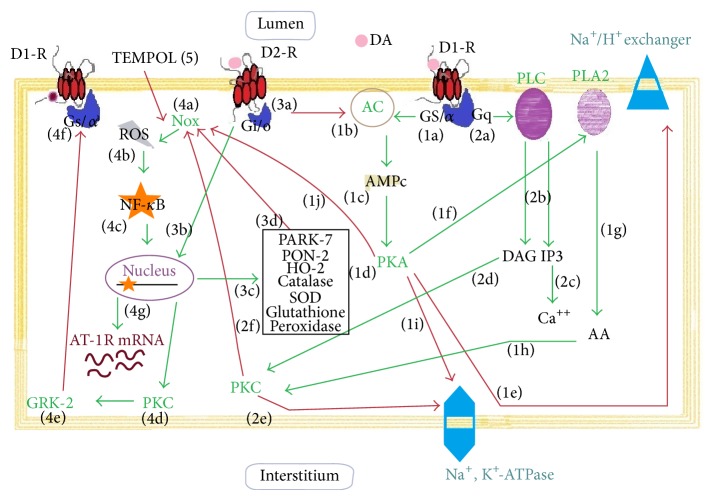
Renal dopamine receptors and its antioxidants properties. Dopamine (DA) binds to D1 receptor (D1R) coupled to stimulatory G proteins (Gs and G*α*) (1a) and to active adenylyl cyclase (AC) (1b) which in turn increases second messenger levels of cAMP (1c) and activates the protein kinase A (PKA) (1d). PKA inhibits directly the Na/K exchanger (1e) in the apical membrane and also stimulates phospholipase A_2_ (PLA_2_) (1f). A metabolite of arachidonic acid (AA) (1g), 20-hydroxyeicosatetraenoic acid (20-HETE) (not shown) activates PKC (1h). In addition, D1R via G protein (Gq) activates PLC (2a) with generation of inositol 1,3,4-trisphosphate (IP_3_) and diacylglycerol (DAG) (2b). IP_3_ induces calcium (Ca^++^) release (2c) and DAG stimulates PKC (2d). Both PKA (1i) and PKC (2e) can inhibit Na^+^, K^+^-ATPase activity (1i) and promote tubular sodium excretion. A crosstalk between PKA (1j) and PKC (2f) results in a negative regulation of NADPH oxidase (Nox) activity. D2 receptors (D2R) are coupled to inhibitory G-protein (Gi/o) (3a) which inhibits AC activity. The D2-R stimulates the expression of antioxidant complex (3b) such as Parkinson protein 7 (PARK-7), paraoxonase 2 (PON2), heme oxygenase 2 (HO-2), catalase, superoxide dismutase (SOD), and glutathione peroxidase (3c). By displaying this antioxidant role, the physiological effect of D2R is to reduce Nox (3d) activity. The activation of Nox (4a) leads to an imbalance in the redox state by generation of reactive oxygen species (ROS) (4b). This conduces to an activation of nuclear factor kappa-light-chain-enhancer of activated B cells (NF-*κ*B) in the cytoplasm and its translocation to the nucleus (4c). NF-*κ*B promotes the transcription of several genes, including PKC (4d) and AT-1 receptor (4g). Activation of PKC stimulates the translocation of G protein-coupled receptor kinase 2 (GRK-2) to the membrane (4e). The GRK-2 causes D1R serine hyperphosphorylation and uncoupling of G-protein to D1 receptor (4f). TEMPOL (5), an antioxidant permeable compound, reduces ROS production and prevents cell damage by oxidative stress. Thin red arrows indicate inhibition. Thick green arrows indicate activation.

Dopamine regulates ROS production in a biphasic manner: at physiological concentrations, dopamine decreases ROS production, via D1-like receptors, but at high concentrations (≥10 *μ*M) it stimulates ROS production [131–133]. Although all dopamine receptor subtypes are capable of decreasing ROS production, only the deletion of D2R or D5R in mice has been associated with increased ROS production [134]. NADPH oxidase activity can be inhibited either by stimulation of D1R, via a PKA and PKC crosstalk, or by direct stimulation of D5R or by indirect inhibition of PLD [134–136]. D5R appears to be more implicated in ROS regulation by dopamine. An experimental study carried out in mice with D5R* knockout* demonstrated a higher level of plasma thiobarbituric acid-reactive substances (TBARS) with high expression and activity of NADPH oxidase [135, 136]. Besides, the D5R may also positively regulate antioxidants as heme oxygenase 1 (HO-1) [137]. All these evidences suggest a major role of D5R for preventing excessive ROS production. Through D1R and D5R, dopamine stimulates antioxidant enzymes like superoxide dismutase (SOD), glutathione peroxidase, glutamyl cysteine transferase, and HO-1 [138–140], resulting in a diminished ROS production and antioxidant protection. The D2R also exhibits antioxidant activity. Endogenous antioxidants are necessary to counterbalance the adverse effects of oxygen free radicals. In this way, Parkinson protein 7 (PARK7 or DJ-1) is a peroxiredoxin-like peroxidase that scavenges H_2_O_2_ through oxidation of Cys-106 and regulates the expression of several antioxidant genes, such as SOD [140–145]. Another antioxidant factor is paraoxonase 2 (PON2), a cell-associated lipolactonase, located in lipid rafts of brush border of proximal tubule that physically interacts with D2R to counterregulate renal NADPH oxidase activity and ROS production [146–148]. Heme oxygenase 2 (HO-2) is another enzyme that degrades heme to generate biliverdin, a metabolite with antioxidant properties [149, 150]. Like D1-like receptors, the stimulation of D2R in the kidney is associated with a decrease of oxidative stress by inhibition of NADPH oxidase expression and increased expression of the endogenous antioxidants DJ-1, PON2, and HO-2, all of which can inhibit NADPH oxidase activity [148, 151]. On the contrary, oxidative stress can affect negatively the function of dopamine receptors. The G-protein receptor kinase (GRK, a serine/threonine kinase) regulates renal D1R and D3R by preventing their reassociation with G proteins, thereby inducing its desensitization [152]. A decrease of intracellular antioxidant factors in proximal tubular cells induces NF-*κ* *β* translocation from the cytoplasm to the nucleus and, subsequently, the upregulation of PKC activity and translocation of GRK to the plasma membrane. In turn, GRK may induce a serine hyperphosphorylation of D1R that leads to uncoupling this receptor from the G-protein, thus impairing its activity (Figure 4) [153–156]. In another study, Gross et al. showed in a model of type 2 diabetes that a dopamine D3R antagonist had a beneficial effect on renal morphology and albuminuria, which was comparable in magnitude with that of ACE-inhibitors treatment. In this model, glomerular and tubulointerstitial expression of TGF-*β*1, a marker of renal fibrosis, was higher in diabetic rats and normalized by the administration of the specific D3R antagonist A-437203 [157]. On the other hand, the increase of TGF-*β*1 expression and its targets Smad3 signaling and Snail1 in human renal proximal tubule cells with D2R single-nucleotide polymorphisms has been reported [158]. Although D1R and D2R are capable of decreasing ROS production, there is still no evidence to consider whether the antifibrotic effect of dopamine could be mediated through suppression of ROS. Future studies should be conducted to clarify this mechanism.

Altogether this evidence permits us to understand the complexity of renal dopaminergic system and opens the possibility of considering dopamine as a potential nephroprotective agent for future therapeutic strategies.

## 5. Renal Interaction between Vasoactive Peptides and the Dopaminergic System

The observation that dopamine and ANP share similar physiological effects suggests that each one may contribute to enhancing the actions of the other. For the past decade, several experimental studies have reported the possibility that natriuretic peptide hormones and the renal dopaminergic system could display a positive interaction. However, beside these interesting observations, the intrinsic mechanisms involved in ANP-dopamine relationship remain unclear and continue to be under investigation. It has been reported that part of the ANP inhibitory effects on sodium and water reabsorption depends on dopaminergic mechanisms, particularly on dopamine receptors [159–164]. ANP can also potentiate the inhibitory effect of dopamine on the Na^+^/H^+^ exchanger in the proximal tubules and can recruit intracellular located D1R to the plasma membrane, showing how ANP facilitates renal dopamine effects [165, 166]. Another study that complements and reinforces this idea was reported by Correa et al., showing that ANP stimulates dopamine uptake by the tubular cells in the kidney, via the stimulation of NPRA receptors, followed by cGMP (as second messenger) and PKG activation. These findings altogether show that ANP favors dopamine intracellular accumulation, which in turn permits D1R recruitment and stimulation, resulting in the overinhibition of Na^+^, K^+^-ATPase activity, the decrease of sodium reabsorption, and the increase of natriuresis [167, 168]. The interaction between circulating ANP and locally formed dopamine in the kidney must be taken into account, since both hormones not only contribute to maintaining a well-balanced regulation of sodium metabolism and blood pressure, but also can potentiate their antioxidant and nephroprotective properties.

There is evidence that dopamine exerts short- and long-term natriuretic effects that oppose Ang II antinatriuretic effects [169, 170]. This interaction between renal dopamine and Ang II takes place mainly at receptor level and this interaction seems to be receptor subtype specific [118]. Dopamine displays sustained effects via D1-like receptors, reducing renal Ang II tonus, decreasing AT1R mRNA and protein expression, and inducing AT2R-dependent natriuresis in the kidney [170]. The first study concerning Ang II effects on renal dopamine uptake concluded that Ang II inhibits dopamine uptake in renal tissues through stimulation of AT1R and signals through the PLC pathway, including the generation of second messengers, IP_3_ and DAG, intracellular calcium release, and activation of PKC and CaM kinase II. These effects altogether may prevent dopamine accumulation into renal tubular cells, resulting in a decrease of dopamine availability inside the cell and at the luminal side of the nephron. These mechanisms may prevent homologous D1R recruitment and stimulation, which in turn would result in disinhibition of Na^+^, K^+^-ATPase activity and the consequently increase of sodium reabsorption and decrease of natriuresis [171–173]. On the other hand, AT2R and D1R oppose each other cooperatively to counteract the vasoconstrictor and antinatriuretic functions elicited by AT1R. It has been demonstrated that* in vivo* administration of fenoldopam (a highly selective D1-like receptor agonist) in sodium loaded Sprague Dawley rats induces the translocation of AT2R from intracellular compartment to the apical plasma membranes [174]. The fact that fenoldopam-induced natriuretic response was completely inhibited by the intrarenal coinfusion of the AT2R antagonist, PD 123390, reinforces this concept [175]. The D3R displays inhibitory effect on renin secretion, while D4R and D5R decrease AT1R expression [176]. D1R as well as D3R inhibit Ang II effects by their heterodimerization with the AT1R [177–179]. In renal proximal tubule cells, D5R can also decrease AT1R expression and its signaling transduction by a c-Src-dependent and proteasome-dependent process [180–182]. ROS can also inactivate D1R and activate AT1R [183]. It has been recently reported that oxidative stress plays a central role in the development of hypertension in old Fischer 344 × Brown Norway F1 rats [184]. In this experimental model, NF-*κ*B diminished renal D1R function and promoted the transcription of AT1R gene, which resulted in high blood pressure levels with aging [184]. Other authors showed that reduction in intrarenal dopamine synthesis is associated with increased detrimental effects of Ang II on renal injury [185, 186]. All these evidences about the interaction between angiotensin and dopamine receptors must be taken into consideration, since both hormones exhibit opposite effects not only on sodium balance and blood pressure, but also on oxidative stress.

## 6. Conclusion and Future Perspectives

Although there is evidence that a defective renal dopaminergic system contributes to the development and maintenance of hypertension, it is still not clear what triggering factor causes the selective defect in the renal dopaminergic system. Some of these trigger factors could be an excess of sodium intake that could lead to an activation of intrarenal Ang II and increase in ROS, an increase in carbohydrate intake and a high fat diet, both factors that promote insulin resistance. Furthermore, the renal dopaminergic system is sensitized by a high salt intake and volume expansion, which opens the question about how intrarenal sodium sensors may influence renal dopamine bioavailability. The observation that dopamine receptors availability in the plasma membrane may be regulated by other hormones, like ANP, could open up a possible therapeutic approach.

A physiological balance between oxidant and antioxidant systems is necessary to maintain the redox equilibrium in a steady state. In the kidney, an increase in oxidative stress is frequently associated with the development of hypertension and renal inflammation.

Therefore, the comprehension of the signaling network triggered by different hormones like ANP, Ang II, and renal dopamine that regulates the production and catabolism of endogenous prooxidant and antioxidant enzymes and modulates the signaling pathways closely related to the oxidative stress will provide a new insight about the pathophysiology of renal inflammation and injury and contribute to improving diagnosis tools, evolution, prognosis, and treatment of renal pathologies.

This could open a new possibility for therapeutic strategies beyond the blockade of RAAS. In this sense, future pharmacological agents targeting maintaining optimal levels of ANP and/or intrarenal dopamine can be proposed as novel nephroprotective agents to prevent renal damage by oxidative stress.

The administration of synthesized analogous of natriuretic peptides (as nesiritide and anaritide) or inhibitors of the catabolic enzyme 24.11 endopeptidase (candoxatril, omapatrilat, neprilysin, and ecadotril) may be a useful tool to regulate the expression of key components of tubulointerstitial inflammation in the kidney and to control the oxygen supply or demand, under circumstances of sodium overload. In a similar sense, the use of antioxidants and Ang II-AT1R blockers (losartan and candesartan) to inhibit the RAAS may contribute to improving the treatment of oxidative stress-derived renal pathologies.
